# Dogs distinguish authentic human emotions without being empathic

**DOI:** 10.1007/s10071-024-01899-x

**Published:** 2024-09-21

**Authors:** Juliane Bräuer, Dorothea Eichentopf, Nomi Gebele, Louise Jandke, Veronique Mann, Katharina Schulte, Yana Bender

**Affiliations:** 1 DogStudies, Max Planck Institute for Geoanthropology, Kahlaische Strasse 10, 07745 Jena, Germany; 2 Department for General Psychology and Cognitive Neuroscience, Friedrich Schiller University of Jena, Am Steiger 3, 07743 Jena, Germany; 3 Institute of Psychology, University of Hamburg, Von-Melle-Park 5, 20146 Hamburg, Germany; 4 IVT — Institut für Verhaltenstherapie GmbH, Markt 22, 07743 Jena, Germany; 5 Vitos Kinder- und Jugendklinik für psychische Gesundheit Eltville, Kloster-Eberbach-Straße 4, 65346 Eltville, Germany

**Keywords:** Domestic dogs, Emotions, Empathy, Social interactions

## Abstract

Several recent studies have investigated how dogs perceive human emotional expressions. They have measured the reactions of dogs when exposed to stimuli presented in different modalities, such as photographs, audio recordings or odor samples, or to humans simulating various emotional situations. In the current study, dog owners were manipulated to genuinely experience emotions of happiness, sadness, and neutrality. We measured how dogs responded to their owners’ authentic emotions in two different natural situations: induction of the emotion through a video clip and training of a new task. Through a detailed analysis of dog behavior in these naturalistic settings, we investigated whether dogs show behavioral responses to genuine human emotions. We found that dogs behaved differently depending on the owner’s emotional state: they gazed and jumped less at owners when they were sad, and their compliance with the ‘sit’ command was also diminished. When owners were happy, dogs performed better in the trained task. These results are discussed in light of how dogs perceive human emotional expressions and the adaptive value of this skill.

**Supplementary Information:**

The online version contains supplementary material available at 10.1007/s10071-024-01899-x.

## Introduction

Dog owners often claim “My dog knows exactly how I feel” (Szánthó et al. 2017). Indeed, it is likely that the ability to perceive and recognize human emotions may have developed in dogs over the long co-evolution process between dogs and humans as it has been adaptive to perceive negative or positive emotions in humans and respond by either avoiding or approaching them (Kujala and Bräuer 2024). A related skill is also thought to exist in dogs’ closest living relatives —wolves, though for a different reason. During hunting, wolves must discern which individuals in a herd of prey to target, and it is advantageous for them to perceive signs of sickness or heightened fear (Bräuer et al. 2017; Gadbois and Reeve 2014). There is also some evidence that wolves are able to recognize the emotional expressions of conspecifics to facilitate social regulation (Maglieri et al. 2024). It is conceivable that this skill of the dog-wolf ancestor was adapted in dogs during the domestication process in order to perceive and predict human behavior.

In recent years, significant attention has been given to the question of whether dogs perceive human emotions and the extent of that understanding. In these studies, dogs are presented with a range of stimuli, including photographs, simulated situations, audio recordings and odor samples of humans and other dogs in different emotional states (see Albuquerque and Resende 2023; Kujala and Bräuer 2024; and Kujala 2017 for reviews). Subsequently, dogs’ reactions to these stimuli are measured. When using facial expressions as stimuli, studies have shown that dogs are able to distinguish negative and positive emotional expressions in human and dog faces (Albuquerque et al. 2016; Barber et al. 2016; Deputte and Doll 2011; Müller et al. 2015; Nagasawa et al. 2011; Somppi et al. 2016; Albuquerque and Resende 2023). For example, dogs learn to distinguish pictures of different human facial expressions, presumably relying on their memories of real emotional human faces to accomplish the task (Müller et al. 2015). Dogs can differentiate between smiling or neutral human faces (Nagasawa et al. 2011) and they gaze longer at smiling or angry faces compared to neutral facial expressions (Hori et al. 2011).

As emotions are not only expressed through facial expressions, other studies involved dogs being presented with humans who simulated emotional situations. For example, owners or strangers either pretended to cry, hummed, or laughed. As a result, dogs orientated toward a person more often when the person was pretending to cry (Custance and Mayer 2012; Meyers-Manor and Botten 2020). In a recent study, actors portrayed different emotions, prompting dogs to exhibit differential behavior depending on the emotional valence of the expression (Souza et al. 2023). Moreover, Van Bourg et al. (2020) investigated whether dogs would release their seemingly trapped owners from a large box depending on their emotional behaviour. Even though dogs were more likely to release owners who called out for help compared to those who read aloud calmly, only a third of all dogs opened the box at all.

However, since pet dogs constantly monitor us, it is likely that they are able to discern between posed and authentic situations involving humans (Bräuer et al. 2013, 2017; Bräuer 2015; Marshall-Pescini et al. 2014). For example, dogs failed to assist when their owners feigned a heart attack (Macpherson and Roberts 2006), probably because they perceived that it was faked (Bräuer 2015; Macpherson and Roberts 2006). Thus, in order to better comprehend the phenomenon of how dogs perceive human emotions, these emotional expressions should be genuine.

To solve this issue, some studies used stimuli featuring authentic emotional situations. For instance, when dogs are presented with either sounds of a human infant crying, babbling, or computer-generated “white noise”, dogs react to the infant crying, displaying a combination of submissiveness and alertness, along with increased cortisol levels (Yong and Ruffman 2014). In a similar setup, three aspects of the presented sounds were varied: emotionality, species, and valence. The authors found that dogs behaved differently after hearing non-emotional sounds compared to emotional sounds, and they could also distinguish between positive and negative valence of the emotion. However, they responded similarly to human and conspecific sounds (Huber et al. 2017; see also Quervel-Chaumette et al. 2016).

Given that dogs have an excellent olfactory sense which they rely on when exploring the environment or recognizing individuals (e.g. Bräuer and Belger 2018), transmission of emotional information via chemosignals is also likely. D’Aniello and colleagues (2018) collected sweat samples from male donors who had watched videos inducing happiness or fear. They found that dogs responded differently to the odor of differing human emotions.

Most studies have investigated only a single aspect or modality of how dogs detect human emotion. Moreover, the perceived emotions were either acted out and not really felt, or the emotional situation was “preserved” through recorded stimuli or an odor dispenser. Thus, in the current study we used a holistic approach in which humans were manipulated to genuinely experience the emotion during the actual experiment. By incorporating visual, auditory and olfactory modalities, dogs may prove to be much more successful in detecting human emotions (Morisaki et al. 2009).

Another more theoretical question arising from the above-mentioned studies is whether dogs show empathy when they react to human emotions. Empathy has several definitions, but is often categorized into emotional (feeling what the other is feeling) and cognitive (understanding the other’s perspective) components by most researchers (see Decety and Ickes 2011 for a review). To explain the underlying processes, Preston and de Waal (2002) introduced the Perception Action Model (PAM). The PAM includes five different classification terms: *emotional contagion*,* sympathy*,* empathy*,* cognitive empathy* and *prosocial behaviors*. The categories differ in the ability (i) to distinguish between self and other, (ii) to be in a matching state and (iii) to actually help the other individual. The present study aims to also address the question of how to classify the dogs’ behavior using the first three classification terms. Previous studies provide evidence for emotional contagion (“The subject’s state results from the perception of human’s state” without helping). However, it remains an open question whether dogs are also capable of sympathy (“The subject feels sorry for the human” without state matching but with helping, Preston and de Waal 2002; see also Decety and Ickes 2011) or empathy (“The subject’s state results from the attended perception of the human’s state” with representational state matching and helping in particular familiar humans, Preston and de Waal 2002; see also Decety and Ickes 2011).

In the present study, our objective was to investigate how dogs react to their owners’ genuine emotional expressions. In contrast to previous studies, we (i) did not focus on one modality in which the human emotion was presented to the dog, and (ii) tested how dogs reacted to their owners’ genuine emotional expressions in a natural situation, both during the induction of the emotion and during a joint dog-owner task. In the induction phase, we used short video clips and a neutral text to induce positive, negative, and neutral emotions in owners, which is a common and effective practice in psychological studies (see Lench et al. 2011 for a review). In the subsequent training phase, the owners were asked to train the dog in a new task. We worked with owners, as their emotions should be most relevant for the dogs. Importantly, the owners were naive about the purpose of the study. By analyzing the dog behavior in these natural situations in detail, we wanted to investigate to what extent and how dogs would distinguish between authentic human emotions.

We hypothesize that dogs’ behaviors differ between the induction phase and the training phase, depending on the owner’s emotional state. Our design allows us to test for within-group comparisons(A) and between-group comparisons (B). (A) Dogs’ behaviors differ between the neutral instruction session and the clip session, when owners were induced with sad and happy emotions. (B) Dogs` behaviors differ in the clip session, depending on the owner’s emotional state. In particular, we expected that if dogs are capable of sympathy or empathy, they should exhibit helpful comforting behaviors, particularly in the sessions where the owners were induced with sad emotions. Thus, they should then stay close to the owner, approach and touch him (Meyers-Manor and Botten 2020; Souza et al. 2023; Quervel-Chaumette et al. 2016; D’Aniello et al. 2018). As this has not been tested before, it is unclear whether we could also expect better obedience then as a result of an increased desire to please when the owner is sad (see Bräuer 2015).

In contrast, we expected less gazing at the owners and more sitting and laying in the neutral situation where everything was normal compared to the emotional situations (Van Bourg et al. 2020). During the sessions in which the owners were induced with happy emotions, we expected more jumping as a reaction to the happy mood of the owner. Regarding the performance in the training task it was again unclear what to expect due to the lack of previous studies.

## Methods

### Subjects

Seventy-nine dogs were tested. To meet inclusion criteria, dogs were required to be proficient in the ‘sit’ command, to be healthy and at least one year old, and the owners had to fully comply with the instructions during the test and not pay attention to the dog in the emotion induction phase. Two dog-owner pairs had to be excluded from the analysis as they did not meet these preconditions. Thus, seventy-seven dogs (Canis familiaris; 47 females and 30 males) of various breeds and ages (range = 1–16 years old, mean age = 5.7 years) successfully participated in this experiment (see Table S1, Supplementary Material). All subjects lived as pet dogs with their owners in Jena, Germany and the surrounding area. The dog owners took part in the study voluntarily and were present during the test. Furthermore, owners did not have prior knowledge of the experiment’s design or the specific scientific questions, they were however provided with information after the last session was completed. After data collection, the dog owners were given access to the videos recorded of themselves and their dogs from the testing session. The test was conducted by three experimenters (KS, DE, VM). The study adhered to the Guidelines for the use of Animals in Research. Approval was obtained from the ethics committee of the Max Planck Society (processing number 2019_17).

### Set up and materials

**Fig. 1:**
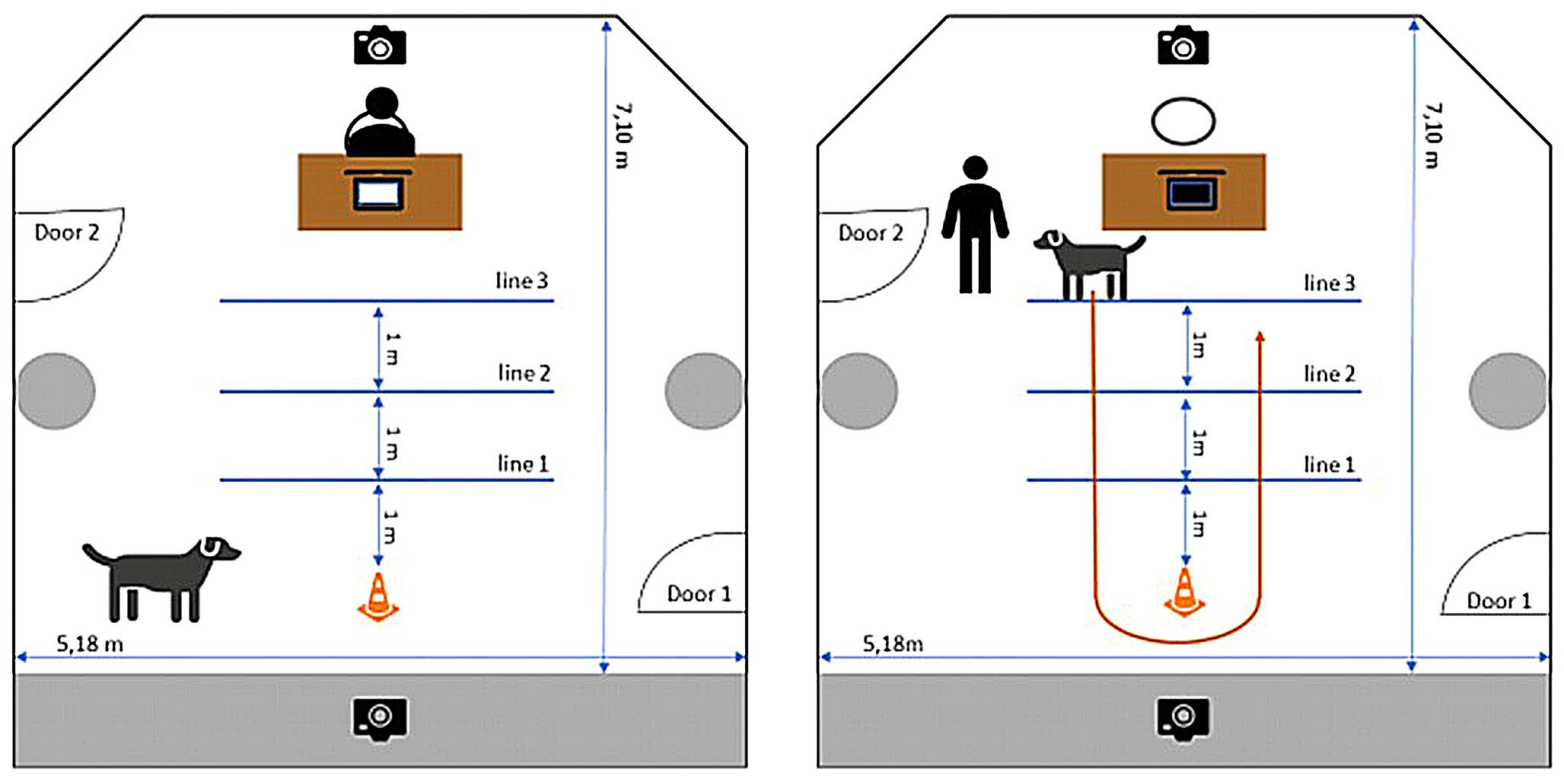
Setup of the study. **a**) induction phase: owner is on the chair, **b**) training phase: arrow indicates the path the dog should learn during training

The experiment took place in a testing room (5.20 m x 7.10 m) at the Doglab of the Max Planck Institute of Geoanthropology (MPI GEA) in Jena, Germany (see Fig. 1). On one side of the room, a chair and a table holding a laptop with headphones were placed. On the opposite site of the room, there was a conical obstacle (30 cm high). Between the table and the cone, three lines were marked on the floor with adhesive tape. These lines were at a distance of 1 m, 2 m and 3 m from the cone, respectively. The entire procedure was recorded with two video cameras from opposite sides of the room.

To induce the respective emotions in dog owners we used the following video clips:

- for the happy group: a film excerpt of ‘Marley & Me’ (length 2:19 min, Frankel 2008).
- for the sad group: a film excerpt of ‘Hachi – A dog’s tale’ (2:51 min, Hallström 2009).
- for the neutral group: an excerpt from the documentation of the WDR ‘Experiment: IQ-Test with wolf and dog’ (2:06 min, Schäfer 2018).

All video clips featured a dog in a central role to increase the emotional responsiveness of the owner. Those videos underwent pretesting in order to ensure they were representative enough to induce the intended emotion. To verify the suitability of the videos for emotion induction, the owners received an emotion scale at the end of the experiment to self-rate their mood. (We could not ask for this after each session in order to keep the owners naive about the study’s hypotheses in terms of emotion recognition.) The owners were asked to assess their mood on a Likert scale from zero (sad) to ten (happy), with the value of five as the middle category indicating a neutral emotion (Lench et al. 2011). Within-group comparisons using the Wilcoxon signed-rank test revealed that the emotions were successfully induced. Owners in the sad group rated their mood lower after the sad clip induction (*M* = 4.41, *SD* = 2.39) than after the neutral induction *M* = 6.93 *SD* = 1.70; *r* = .71, *p* < .001). Similarly, owners in the happy group rated their mood higher after the happy clip induction (*M* = 7.65, *SD* = 1.76) than after the neutral induction *M* = 6.38, *SD* = 1.63; *r* = .64, *p* = .002). In contrast, there was no significant difference for owners in the neutral group between neutral instruction induction (*M* = 7.23, *SD* = 1.80) and neutral clip induction (*M* = 6.88, *SD* = 1.98; *r* = .24, *p* = .217. Moreover, owners in the sad group rated their mood lower after the sad induction compared to owners in the happy group after the happy induction (Mann–Whitney U = 87.50; *N1* = 27; *N2* = 24; *r* = .63; *p* < .001). This confirms that the respective stimuli induced genuine sad and happy emotions in our sample (for details see Fig. S1 in Supplementary Materials).

## Procedure

The experiment consisted of two sessions, that were presented one after the other on the same day. Each session was divided into two phases: the emotion induction phase and the training phase (for details see Table 1). The first session was the *Instruction Session.* In the first phase of the Instruction Session, owners were asked to read the detailed instructions from the experimenter to induce a neutral emotion (*Instruction Induction*). In the second phase of the Instruction Session the owners were asked to teach their dog to walk around the cone (*Instruction Training*).

The second session was the *Clip Session*. In the first phase of this session, owners were asked to watch a video, which induced either a happy, sad, or again a neutral emotion (*Clip Induction*). Participants were randomly assigned one of three different clips, either the neutral, the sad or the happy clip (see above). In the second phase of the Clip Session, owners were once again asked to teach their dog to walk around the cone (*Clip Training*).

At the start of the experiment, the owner, dog and experimenter entered the testing room (see Fig. 1). The experimenter explained the procedure, while the dog was allowed to explore the room. The experimenter informed the owner that the study’s purpose was to train the dog to walk from one of the lines around the cone. The owner was also instructed how they should behave in the two phases. The experimenter then left the testing room. The owner sat down on the chair at the desk and read the detailed instructions (see Supplementary Materials for details) from a sheet of paper. To keep this process neutral, we aimed not to induce emotions (neutral situation), which was also confirmed when owners filled out the emotion scale (see above). While reading the instructions owners were not allowed to react to the dog who could freely explore the room. This was the Instruction Induction Phase.

After the owner finished reading, they immediately had to start training the trick. The trick involved the dog sitting on one of the lines close to the owner, going around the cone alone, and returning to the owner at the line (see Supplementary Materials for details). If the dog succeeded in performing this trick from the first line close to the cone three times in a row, the owner had to train them from the second and the third line (Instruction Training). After three minutes of training, the experimenter re-entered the testing room, indicating that the training was over, no matter how successful it was. This was the Instruction Training Phase. The success of the dog-owner pairs varied but no pair managed to solve the most difficult version of the task: that the owner waited on line 3 while the dog walked around the cone (see Fig. 1).

The experimenter then immediately informed the owner it was time for the break and opened one of the three video clips on the laptop for the owner to watch. After that, the experimenter left the testing room. While watching the video clip, the owner again was instructed to completely ignore the dog, allowing them to move freely in the room. This was the Clip Induction Phase. Once the video clip concluded, the owner had to resume the training in exactly the same way as before for another three minutes. This was the Clip Training Phase.

After both sessions, the experimenter asked the owner to fill out the emotion scale to verify the successful emotion induction (0 = sad to 10 = happy, see above). After that, the experiment concluded and the experimenter informed the owner about the true purpose of the study.

**Table 1:** Details of the procedure and variables that were coded in each task (*depending on how long it took the owners to read the instructions)

Session	Phase	Length coded	Group Happy	Group Sad	Group Neutral	Measures
Instruction Session	Instruction Induction	Varied*	Neutral	Neutral	Neutral	Gaze: frequency (%) Touch: frequency (%) Approach: frequency (%) Lay/sit: duration (%) Distance close/medium (%)
	Instruction Training	180 s				Gaze: frequency Touch: frequency Jump: occurence Sit obeyed: frequency (%) Overall success
Clip Session	Clip Induction	120 s	Happy Clip	Sad Clip	Neutral Clip	Gaze: frequency (%) Touch: frequency (%) Approach: frequency (%) Lay/sit: duration (%) Distance close/medium (%)
	Clip Training	180 s				Gaze: frequency Touch: frequency Jump: occurrence Sit obeyed: frequency (%) Overall success

## Coding and data analysis

The behavioral variables were coded from the videos (see Tables 1 and 2). The variables were defined based on observed shown behavior, considering previous studies (Van Bourg et al. 2020; Huber et al. 2017; Quervel-Chaumette et al. 2016). Vocalization was also coded, but the results are not presented here due to their infrequent occurrence. For organizational reasons, a total of four primary coders coded the material, each coding all dependent variables for a set of dogs. In order to assess the reliability of the observational data, a fifth independent observer, naïve to the purpose of the study, coded 20% of randomly selected trials. Inter-observer agreement with this naïve coder was excellent and exceeded 0.70 for all included measures. Table 2 summarizes the definition for each measure and the inter-observer reliability. Cohen’s Kappa was used for nominal data, and Intraclass Correlation Coefficients using a two-way random effects model for metric data.

**Table 2:** Definition of the coded variables and inter-observer reliability for each measure

Phase	Measure	Definition	Inter-observer reliability
Induction	Gaze: frequency	Dog looks directly into owner’s face	*ICC* = 0.80***, *N* = 30
	Touch: frequency	Dog touches owner with at least one part of its body	*ICC =* 0.87***, *N* = 30
	Approach: frequency	Dog walks into predetermined ‘close’area around human with at least one paw (see Fig. S2, Supplementary Materials)	*ICC* = 0.91***, *N* = 30
	Lay/sit: duration	Time dog spends sitting or laying down	*ICC* = 0.97***, *N* = 30
	Distance close/medium	All four of the dog’s paws are in area within max. 3 m distance of where the owner is sitting (see Fig. S2, Supplementary Materials)	*ICC* = 0.93***, *N* = 30
Training	Touch: frequency	Dog touches owner with at least one part of its body	*ICC* = 0.73***, *N* = 30
	Jump: occurrence	Dog lifts both front legs, at least one paw has contact to the owner	Kappa = 0.93***, *N* = 30
	Sit obeyed: frequency	Dog sits down within a maximum of two seconds after the “sit” command	*ICC* = 0.87***, *N* = 30
	Gaze: frequency	Dog looks directly into owner’s face	*ICC* = 0.70**, *N* = 30
	Overall success: frequency	Number of times within one training phase that owner and dog complete the task (i.e. owner stands at least behind line 1, dog walks from that line and rounds the pylon without being pushed or touched by the owner)	*ICC* = 0.95***, *N* = 30

*Note* *** = *p* < .001, ** = *p* < .01, * = *p* < .05

The software *SPSS* was used for all analyses. We conducted a Shapiro-Wilk test, which showed that the data significantly deviated from normal distribution. Therefore, all statistical tests were non-parametric, two-tailed and the alpha level was set to 0.05. We used the Wilcoxon signed-rank test for continuous and the McNemar test for categorical variables in the within-group comparison. We applied the Kruskal-Wallis test for continuous variables and the Pearson chi-square test (or the fisher’s exact test, if any expected cell frequencies were less than 5) for nominal variables in the between-group comparison of the three emotional stimuli.

## Results

### Within-Group comparison

**Fig. 2:**
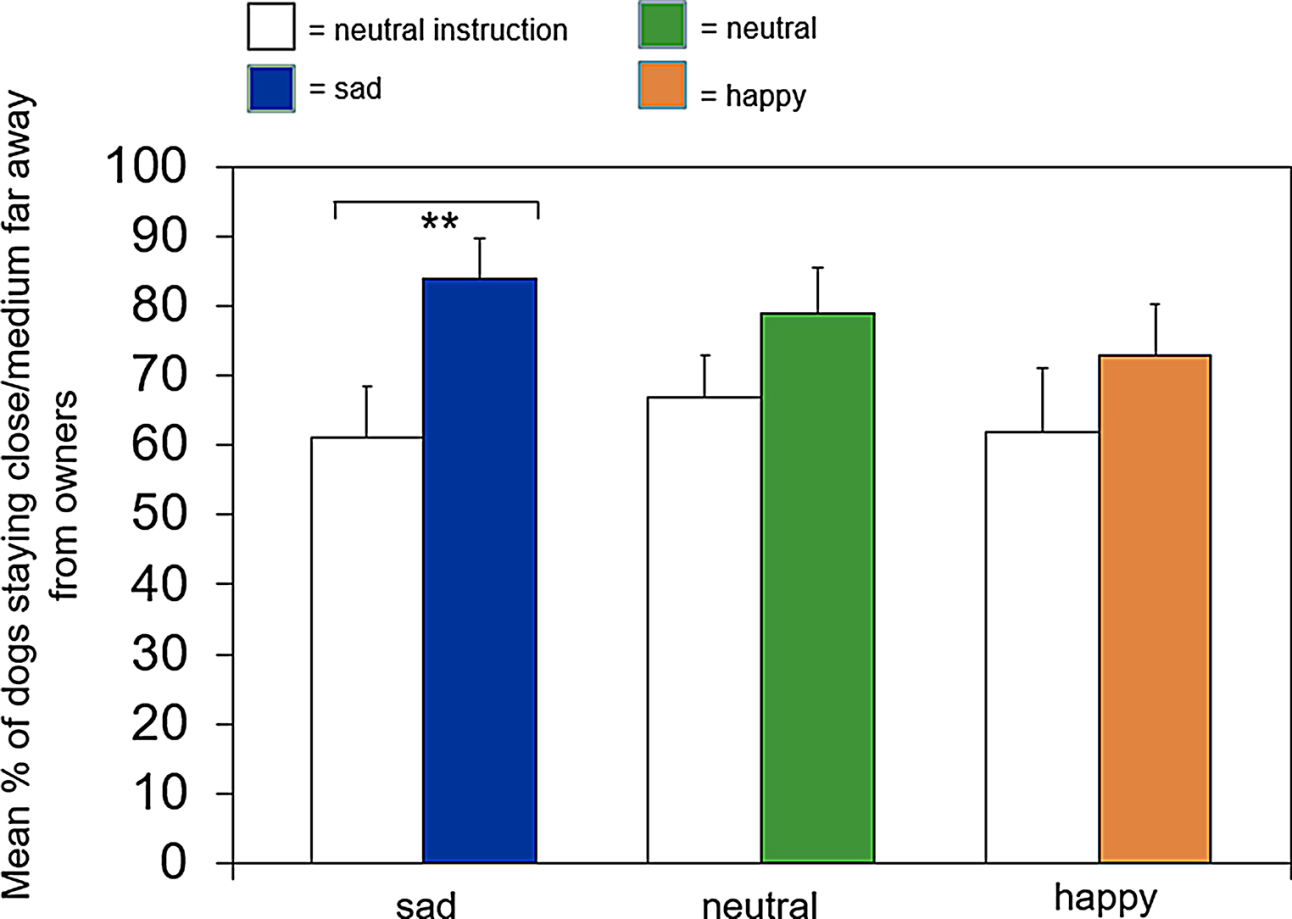
Comparison of mean percentage (+/- SE) of how long dogs stayed close or medium far from the owners for the sad, happy and neutral clip induction phase, compared to the neutral instruction induction phase

The within-group comparison aimed to test if dogs’ behaviors differed between the Instruction Session and the Clip Session when owners were induced with sad and happy emotions. An overview of all analyzed variables for the induction phases can be found in Table 3. As shown in Fig. 2, in the sad clip induction phase, dogs stayed longer in close proximity to their owners (*r* = .59, *p* = .001) compared to the introduction induction phase.

Dogs sat and laid down more in all clip induction phases, compared to the instruction induction phase (sad: *r* = .61, *p* = .001, happy: *r* = .56, *p* = .005, neutral: *r* = .73, *p* < .001). This strongly suggests an underlying order effect. Dogs furthermore gazed less at their owners in the neutral clip induction phase (*r* = .56, *p* = .006), compared to the neutral introduction induction phase. Interestingly, that was not the case in the groups that were induced with happy or sad emotions. This implies that dogs in a neutral situation in the second session gazed less at the owner, as they got used to the testing situation (order effect). However, when owners were induced with emotions, there was no difference in gazing at the owner between emotion induction phase and introduction induction phase, indicating that dogs in these phases looked more at the owners. In contrast, no significant differences were found within the groups for the variable *touch* and *approach.*

**Table 3:** Results of the within-group comparison for the induction phases using the Wilcoxon signed-rank test

Variable	Comparison	Coding	*M*	*SE*	*R*	*P*
Gaze	sad vs. neutral instruction	Frequency	Sad: 2.10 neutral instruction: 2.76	Sad: 0.08 neutral instruction: 0.12	0.15	0.469
Gaze	happy vs. neutral instruction	Frequency	Happy: 2.30 neutral instruction: 3.62	Happy: 0.59 neutral instruction: 0.85	0.22	0.200
Gaze	neutral vs. neutral instruction	Frequency	Neutral: 2.46 neutral instruction: 6.74	Neutral: 0.46 neutral instruction: 1.40	0.56	**0.006**
Touch	sad vs. neutral instruction	Frequency	Sad: 0.18 neutral instruction: 0.69	Sad: 0.07 neutral instruction: 0.22	0.38	0.057
Touch	happy vs. neutral instruction	Frequency	Happy: 0.24 neutral instruction: 0.81	Happy: 0.10 neutral instruction: 0.55	0.12	0.641
Touch	neutral vs. neutral instruction	Frequency	Neutral: 0.53 neutral instruction: 1.01	Neutral: 0.20 neutral instruction: 0.64	0.03	0.920
Approach	sad vs. neutral instruction	Frequency	Sad: 0.77 neutral instruction: 1.36	Sad: 0.13 neutral instruction: 0.32	0.27	0.163
Approach	happy vs. neutral instruction	Frequency	Happy: 0.92 neutral instruction: 1.64	Happy: 0.21 neutral instruction: 0.56	0.30	0.156
Approach	neutral vs. neutral instruction	Frequency	Neutral: 0.95 neutral instruction: 1.76	Neutral: 0.22 neutral instruction: 0.61	0.22	0.329
Lay/sit	sad vs. neutral instruction	Duration	Sad: 53.95 neutral instruction: 26.68	Sad: 8.33 neutral instruction: 7.55	0.61	**0.001**
Lay/sit	happy vs. neutral instruction	Duration	Happy: 55.73 neutral instruction: 33.37	Happy: 8.43 neutral instruction: 8.99	0.56	**0.005**
Lay/sit	neutral vs. neutral instruction	Duration	Neutral: 39.73 neutral instruction: 9.07	Neutral: 8.16 neutral instruction: 4.62	0.73	**<0.001**
Distance close/ medium	sad vs. neutral instruction	Duration	Sad: 81.79 neutral instruction: 60.84	Sad: 5.74 neutral instruction: 7.25	0.59	**0.001**
Distance close/ medium	happy vs. neutral instruction	Duration	Happy: 71.32 neutral instruction: 63.35	Happy: 7.91 neutral instruction: 8.83	0.30	0.168
Distance close/ medium	neutral vs. neutral instruction	Duration	Neutral: 75.79 neutral instruction: 79.32	Neutral: 6.67 neutral instruction: 7.08	0.41	0.053

*Note* Significant results are in bold

**Fig. 3:**
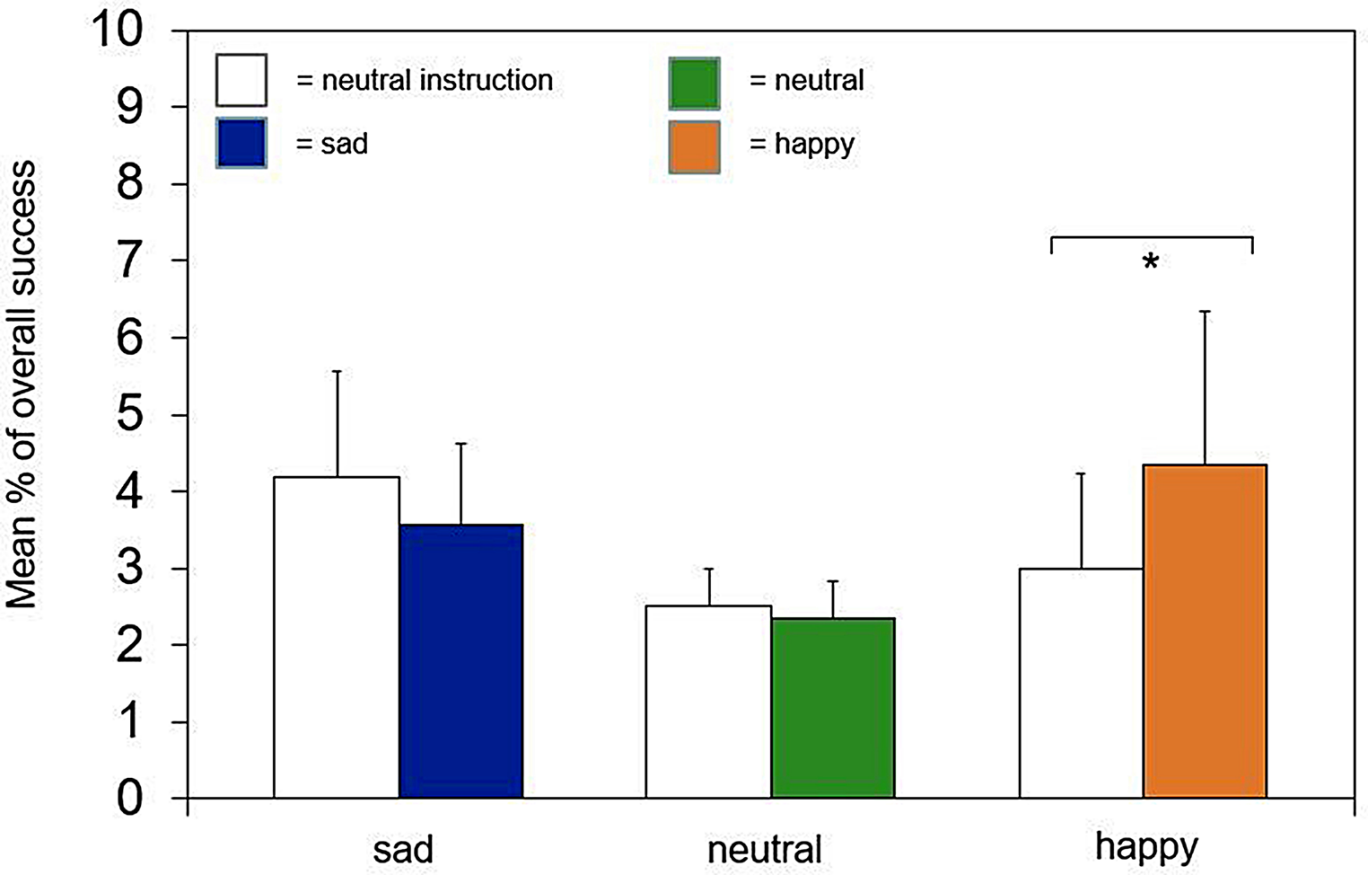
Comparison of mean overall success (+/- SE) between Instruction Training and Clip Training for all three groups

Table 4 contains all analyzed variables for the training phases, with only significant relationships reported as follows. As shown in Fig. 3, dogs performed better in the trained task (overall success) after owners saw the happy clip compared to the training following the neutral instruction (*r* = .46, *p* = .036). Dogs also gazed less at their owners (*r* = .38, *p* = .048) and exhibited poorer obedience to the sit command (*r* = .42, *p* = .035) in the sad clip training compared to the neutral instruction training. No significant differences within the groups were found for the variables *touch* and *jump* in the training phases.

**Table 4:** Results of the within-group comparison for the training phases using the Wilcoxon signed-rank test for continuous and the McNemar test for categorical variables

Variable	Comparison	Coding	*M*	*SE*	*R*	*P*
Touch	sad vs. neutral instruction	Frequency	Sad: 14.07 neutral instruction: 14.04	Sad: 2.06 neutral instruction: 2.30	0.02	0.910
Touch	happy vs. neutral instruction	Frequency	Happy: 13.09 neutral instruction: 12.87	Happy: 1.86 neutral instruction: 2.20	0.12	0.556
Touch	neutral vs. neutral instruction	Frequency	Neutral: 15.58 neutral instruction: 16.19	Neutral: 1.99 neutral instruction: 1.90	0.07	0.741
Jump	sad vs. neutral instruction	Occurrence	Sad: 0.30 neutral instruction: 0.33	Sad: 0.09 neutral instruction: 0.09	0	1.000
Jump	happy vs. neutral instruction	Occurrence	Happy: 0.30 neutral instruction: 0.30	Happy: 0.10 neutral instruction: 0.10	0	1.000
Jump	neutral vs. neutral instruction	Occurrence	Neutral: 0.62 neutral instruction: 0.39	Neutral: 0.10 neutral instruction: 0.10	0.34	0.180
Sit obeyed	sad vs. neutral instruction	Frequency	Sad: 6.04 neutral instruction: 6.44	Sad: 0.80 neutral instruction: 0.76	0.42	**0.035**
Sit obeyed	happy vs. neutral instruction	Frequency	Happy: 5.04 neutral instruction: 5.26	Happy: 1.08 neutral instruction: 1.09	0.32	0.135
Sit obeyed	neutral vs. neutral instruction	Frequency	Neutral: 6.92 neutral instruction: 7.54	Neutral: 0.73 neutral instruction: 0.71	0.26	0.179
Gaze	sad vs. neutral instruction	Frequency	Sad: 24.67 neutral instruction: 26.85	Sad: 1.74 neutral instruction: 1.87	0.38	**0.048**
Gaze	happy vs. neutral instruction	Frequency	Happy: 22.13 neutral instruction: 23.09	Happy: 1.86 neutral instruction: 1.84	0.26	0.206
Gaze	neutral vs. neutral instruction	Frequency	Neutral: 22.96 neutral instruction: 23.15	Neutral: 1.63 neutral instruction: 1.59	0.04	0.857
Overall success	sad vs. neutral instruction	Frequency	Sad: 3.56 neutral instruction: 4.19	Sad: 1.07 neutral instruction: 1.37	0.24	0.232
Overall success	happy vs. neutral instruction	Frequency	Happy: 4.35 neutral instruction: 3.00	Happy: 2.00 neutral instruction: 1.23	0.46	**0.036**
Overall success	neutral vs. neutral instruction	Frequency	Neutral: 2.50 neutral instruction: 2.35	Neutral: 0.50 neutral instruction: 0.48	0.07	0.742

*Note* Significant results are in bold

### Between-group comparison

We conducted a between-group comparison to test the hypothesis whether dogs’ behavior differed in the clip session depending on the owner’s emotional state. In the Clip Induction phase, none of the variables (*gaze*,* touch*,* approach*,* lay-sit*,* distance close/medium*) showed significant differences in the comparison between the three groups (see Table S2 Supplementary Materials).

**Fig. 4:**
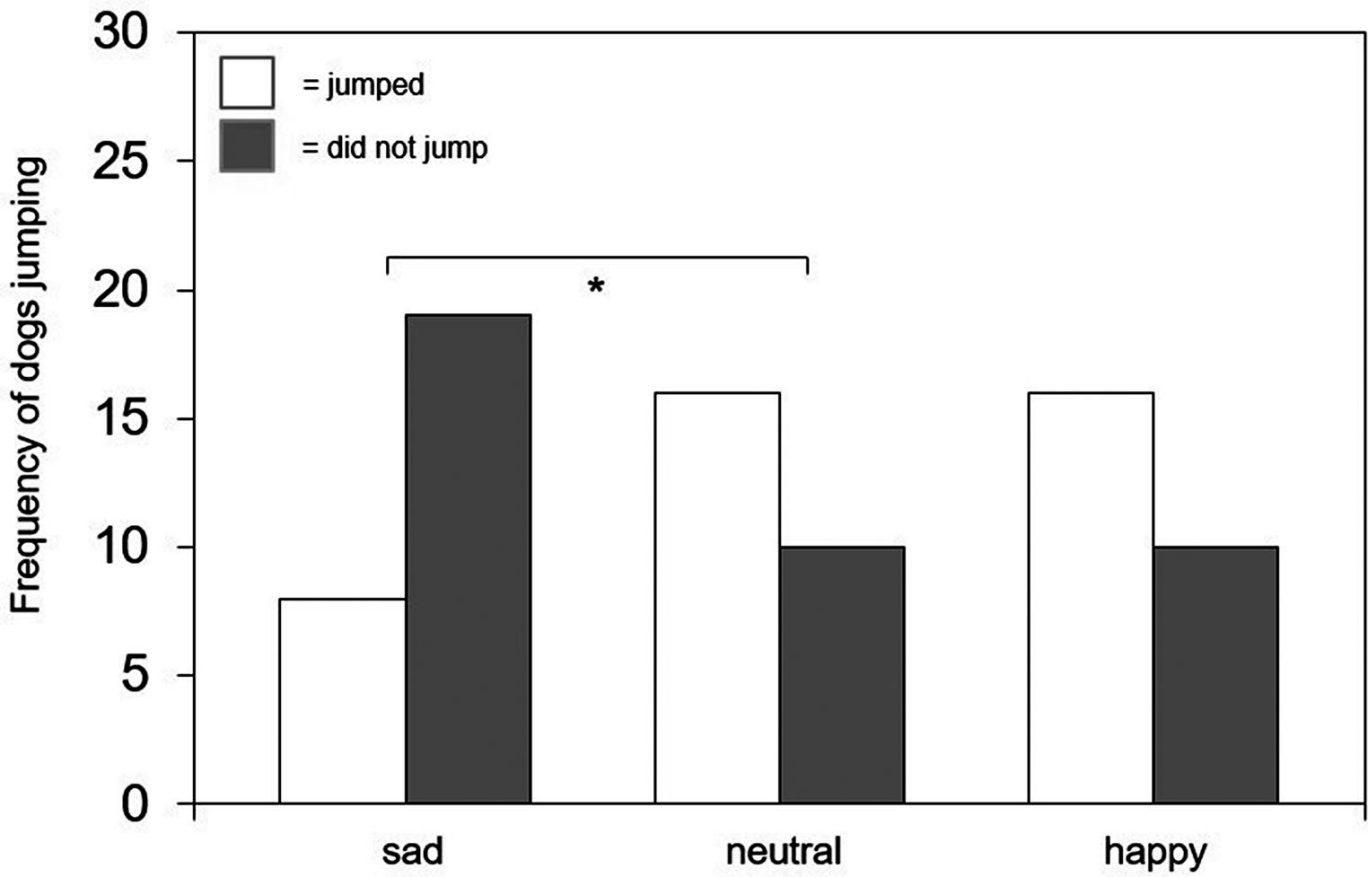
Number of dogs that jumped and did not jump in the sad training phase, compared to the neutral training phase

In the Clip Training phase, a significant difference between the three groups was found for the jump variable (*v* = 0.29, *p* = .038). As illustrated in Fig. 4, further chi-square tests revealed that dogs jumped up on the owner more often in the neutral clip training compared to the sad clip training (*v* = 0.32, *p* = .020). All other variables (*touch*,* sit obeyed*,* gaze*,* overall success*) showed no significant differences between the neutral, sad and happy clip training (see Table S3 Supplementary Materials).

## Discussion

To investigate emotion recognition, we exposed dogs to their owners in genuine emotional states in a natural setting. Consistent with previous research and confirming our main hypothesis, we found that dogs behaved differently depending on the owner’s emotional state (Kujala and Bräuer 2024; see above). Although owners stated that the emotions were successfully induced, we did not notice any obvious behavioral changes in the owners between sessions. Indeed, they themselves believed that the focus of the study was on how to train their dog to perform the task. Thus, it is very likely that dogs’ behavior was actually influenced by the emotional state of the owner.

One related question is whether dogs are capable of sympathy or empathy (Huber et al. 2017; Quervel-Chaumette et al. 2016), although we could not test this directly with the setup of the current study. As posited in our hypothesis, dogs should exhibit helpful and comforting behaviors, particularly when owners are induced with sad emotions. Indeed, when owners were sad, dogs spent more time in the half of the room where the owner was, compared to a neutral situation. We also found evidence that dogs looked at their owners when sad (or happy) emotions were induced whereas they lost interest looking at them when no emotions were induced. However, dogs did not approach or touch owners more often in the emotional situations. Moreover, during the training of the task, dogs gazed at their owners less and jumped on them less when they were sad. Based on these observations, dogs seem to avoid owners when they are sad rather than comfort them. Thus, dogs can clearly distinguish the emotion of sadness, and they even seem to try to take advantage of it, as they were less likely to obey the sit command when owners were sad. This suggests that dogs may perceive that the owner is somehow distracted and therefore less likely to intervene when the dog does not follow commands (see Virányi et al. 2004).

In addition, we found an interesting effect when owners were induced with a happy emotion: dogs performed better in the trained task. One possible explanation might be that the positive mood of the owner is transferred to the dog, which enhances cooperation and, consequently, improves the performance in the task. Another, not mutually exclusive, explanation is that the owner while being happy becomes a more effective trainer for the task.

According to our findings and considering our methodological approach, it appears unlikely that dogs are capable of *sympathy* or *empathy*. If dogs felt sorry for their owners without state matching (i.e. *sympathy*), they should have displayed some form of support for their owners when they were sad, such as approaching them. We also found no evidence for *empathy* which would require a clear distinction between self and other, as well as the ability to react to the situation, for example by helping the emotional individual (Kujala and Bräuer 2024; Lench et al. 2011). In other studies, it was shown that dogs are motivated to help humans without a reward, for instance by opening a door for them (Van Bourg et al. 2020; Bräuer et al. 2013). Crucially, however, dogs must understand the situation and how they can help (see Bräuer 2015 for a review). Sanford and colleagues (2018) investigated whether dogs, in addition to being attentive to the human emotion, also provide help. In the study, the owner, who was either pretending to cry or humming, was trapped behind a door. It was tested whether the dogs would open the door for their owners. The results were not entirely conclusive, as only half of the dogs opened the door for their owners, regardless of whether owners were crying or humming. However, the dogs opened the door more quickly when the owners pretended to cry. It is worth mentioning that some dogs in that study reacted even when the owners only pretended to cry.

In the current study, the human emotions were genuine, and dogs evidently could distinguish between the emotional states of the owner. However, there was no obvious way for the dogs to react in a manner that would help to solve the owner`s problem. Therefore, dogs might have perceived the situation as “there is something wrong with my owner, I better stay at a certain distance, but also not too far away”. Thus, we could show that dogs perceive the different emotions of the human, but it is unclear whether we can talk of *emotional contagion*, i.e., the transference of emotions (Preston and de Waal 2002). Findings of other studies support the explanation that a dog’s state results from its perception of the owner’s state: when confronted with a negative emotion of a human or another dog, they exhibited submissiveness, alertness, increased cortisol levels, more stressful behaviors, and higher heart rates (Kujala and Bräuer 2024).

One related question that arises is how dogs learn to perceive human emotions and whether it is limited to their owner. It is very likely that the tested dogs have seen their owners in a sad (or happy) mood many times before, and may have learned that it is better to keep away from an unhappy owner. In a study by Merola and colleagues (2014), a box with an emotional message was delivered by either the owner or a stranger. Dogs chose to investigate a box eliciting an expression of happiness rather than fear or neutrality in their owner. In contrast, they had difficulties differentiating the boxes delivered by a stranger. Furthermore, Custance and Mayer (2012) found that dogs approached owners and strangers differently when they pretended to cry. They behaved submissively toward the owner, while they sniffed, nuzzled, and licked the stranger in that situation (Custance and Mayer 2012). However, it remains unclear whether dogs react differently to their owners because they have experience with them in emotional situations or because they have a closer relationship to them. There is some evidence supporting the latter possibility. In the aforementioned study by Sanford and colleagues (2018), they also evaluated the bond between dog and owner. Their results suggest that dogs who open the door for their crying owners may have a stronger bond with their owner than those who do not open the door.

In conclusion, dogs are clearly able to perceive genuine human emotions, in particular those of their owners. This unique sensitivity might be adaptive for dogs. For instance, they can utilize emotional information to find food, as seen in a study where the human reacted emotionally (happy, neutral or disgusted) to the hidden contents of two boxes, and dogs chose the box that the human pretended to be happy about (Buttelmann and Tomasello 2013). Additionally, dogs can use their sensitivity to humans to learn about potentially dangerous objects through social referencing (Merola et al. 2012a, b). Similar to children, dogs seek information about an object from the owner. When owners are anxious, dogs inhibit their movements toward the object. Conversely, if owners are relaxed with the object, dogs move toward it and interact with it sooner (Merola et al. 2012a, b). A recent study investigated how dogs witnessed a neutral, positive or negative interaction between two unfamiliar humans. One person always played the neutral role of the giver, while the other one was the receiver, who reacted with different facial expressions to received objects. After witnessing these interactions, dogs could approach a food resource that varied in accessibility. Dogs showed a clear preference for the actor who displayed the more positive emotion, regardless of whether that person was the giver or the receiver. The authors concluded that dogs are capable of accessing implicit information from the humans’ emotional states and use this affective information to make context-dependent decisions (Albuquerque et al. 2022).

Dogs’ sensitivity to human emotions and well-being is not only beneficial to themselves but is also adaptive for humans. They can alert patients experiencing epilepsy and diabetes to seizures, and might even be able to predict such events, even without specific training (Catala et al. 2019; Dalziel et al. 2003; Lim et al. 1992). While dogs may not be empathic, their tendency to constantly monitor humans (Bräuer et al. 2014), their ability to perceive when something is wrong with the owner and their motivation to help (Bräuer et al. 2015) can be highly valuable for humans and should be further investigated.

## Electronic supplementary material

Below is the link to the electronic supplementary material.

Supplementary Material 1

Supplementary Material 2

Supplementary Material 3

Supplementary Material 4

Supplementary Material 5

## Data Availability

The author confirms that all data generated or analysed during this study are included in this published article.
