# Supplementary figures and images for: Dogs distinguish authentic human emotions without being empathic
Source: Anim Cogn. 2024 Sep 21;27(1):60. doi: 10.1007/s10071-024-01899-x (PMC11416375; doi:10.1007/s10071-024-01899-x)

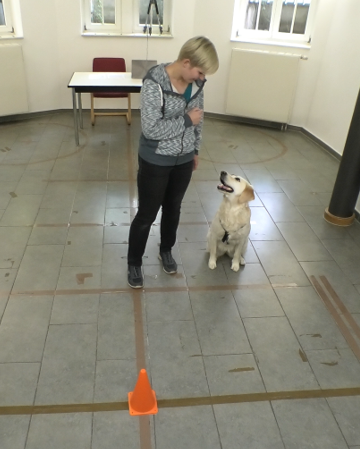

Supplement: Supplementary file 1 — Supplementary Material 1 [file 10071_2024_1899_MOESM1_ESM.png]
