# Supplementary material for: Dogs distinguish authentic human emotions without being empathic
Source: Anim Cogn. 2024 Sep 21;27(1):60. doi: 10.1007/s10071-024-01899-x (PMC11416375; doi:10.1007/s10071-024-01899-x)
Supplement: Supplementary file 4 — Supplementary Material 4 [file 10071_2024_1899_MOESM4_ESM.docx]

**Methods**

*Subjects*

**Table S1: Detailed information about the participating dogs.**

| **Condition Dog’s name** (first syllables) | **Breed**  **Age Sex** | | | | | |
| --- | --- | --- | --- | --- | --- | --- |
| happy As Fu Mix | | |  | 3 | f |  |
| As Ge Husky | | |  | 11 | m |  |
| Be Hey Samojede | | |  | 4 | f |  |
| Bi Ehr Golden Retriever | | |  | 10 | m |  |
| Bi Schwa Labrador | | |  | 4 | m |  |
| Ca He Irish Setter | | |  | 7 | m |  |
| Co Wi Bavarian Mountain  Scent Hound | | |  | 9 | f |  |
| Da Bo French Bulldog | | |  | 4 | f |  |
| Da Rüm Miniature Schnautzer | | |  | 4 | f |  |
| Em Phi Pug | | |  | 3 | f |  |
| Em Wi Lagotto Romagnolo | | |  | 6 | f |  |
| Fe Thu Beagle | | |  | 12 | m |  |
| Fr Bi Small Munsterlander | | |  | 12 | f |  |
| Ja De Golden Retriever | | |  | 4 | m |  |
| Li Schr Gordon Setter | | |  | 9 | f |  |
| Li Si Mix | | |  | 8 | m |  |
| Lu Gla Border Collie | | |  | 8 | f |  |
| Lu Ma Schafspudel | | |  | 4 | f |  |
| Na Br Mix | | |  | 6 | f |  |
| Ne Bo German Wirehaired Pointer | | |  | 2 | m |  |
| Pe Lo Bracco Italiano | | |  | 5 | f |  |
| Qui He Mix | | |  | 1 | m |  |
| So Ko Border Collie | | |  | 1 | f |  |
| Sp Sk Mix | | |  | 7 | m |  |
| Ti Ge Tibetan Terrier | | |  | 3 | m |  |
| Xen Falk Dachshund | | |  | 6 | f |  |
| sad Ai Ep Eurasier | | |  | 2 | m |  |
| Cha Er Australian Shepherd | | |  | 2 | m |  |
| Co He Labrador Retriever | | |  | 3 | f |  |
| Da Ge Mix | | |  | 7 | m |  |
| a Ed Ul Belgian Malinois | | |  | 1 | f |  |
| Em Hue Golden Retriever | | |  | 6 | f |  |
| a Fe Da Old German Shepherd | | |  | 0.5 | m |  |
| Fe Sche Mix | | |  | 9 | f |  |
| Fi Phi Mix | | |  | 3 | f |  |
| Fr Si Mix | | |  | 3 | f |  |
| Hi Schi Wire Fox Terrier | | |  | 1 | f |  |
| Ho Ge German Shepherd | | |  | 5 | m |  |
| Jo Ho Altdeutscher Schäferhund | | |  | 4 | f |  |
| Ki He Labrador Retriever | | |  | 10 | f |  |
| Li Fu Border Collie | | |  | 6 | f |  |
| Lu Ju Mix | | |  | 8 | m |  |
| Lu Pe Golden Retriever | | |  | 1 | f |  |
| Lu Toell Jack Russell Terrier | | |  | 6 | f |  |
| Ma Ge Mix | | |  | 9 | f |  |
| Na Wi Mix | | |  | 5 | f |  |
| Na Ca Tibet Spaniel | | |  | 3 | m |  |
| Pe Wa Mix | | |  | 7 | m |  |
| Ta Sche Russian Tsvetnaya Bolonka | | |  | 5 | m |  |
| Ted Dech Labrador Retriever | | |  | 5 | m |  |
| Vi Frei Chihuahua | | |  | 5 | f |  |
| Will Spi Golden Retriever | | |  | 6 | m |  |
| Yo He Border Terrier | | |  | 2 | m |  |
| Ze Schr Irish Setter | | |  | 10 | m |  |
| neutral An Hill Mix | | |  | 6 | m |  |
| Ba Ko Bouvier des Flandres | |  | | 4 | m |  |
| Big Kra Labrador | |  | | 3 | m |  |
| Bja Ek Deutscher Schäferhund | |  | | 16 | m |  |
| Ca Bie Hovawart | |  | | 10 | f |  |
| Ca Moh Malinois | |  | | 7 | m |  |
| Dex Krus Chihuahua | |  | | 13 | m |  |
| El Heu Boston Terrier | |  | | 7 | f |  |
| El Koch Magyar Viszla | |  | | 2 | f |  |
| Fie Rie Magyar Viszla | |  | | 1 | m |  |
| Fie Sch Airedale Terrier | |  | | 3 | m |  |
| Fi Ro Golden Retriever | |  | | 4 | m |  |
| Her Fül German Sheperd | |  | | 3 | m |  |
| Hjal Lo Mix | |  | | 14 | m |  |
| I Trie Labrador | |  | | 3 | f |  |
| Jack Ha Parson Jack Russell Terrier | |  | | 14 | f |  |
| Karl Sch Pug | |  | | 7 | m |  |
| Lai Vogt Labrador | |  | | 1 | f |  |
| Lu Kraus Labrador | |  | | 2 | f |  |
| Ma Bö – Sch Elo | |  | | 4 | f |  |
| Mo Ahl Labrador | |  | | 1 | f |  |
| Ne Hopf Miniature Poodle | |  | | 8 | m |  |
| Ni Heit Cavalier King Charles Spaniel | |  | | 8 | m |  |
| Quin Heß Welsh Terrier | |  | | 12 | m |  |
| Se Be Mix | |  | | Unkown | m |  |

*Note.* Dogs of more than one breed or of unknown decent are referred to as “Mix”.
a Excluded


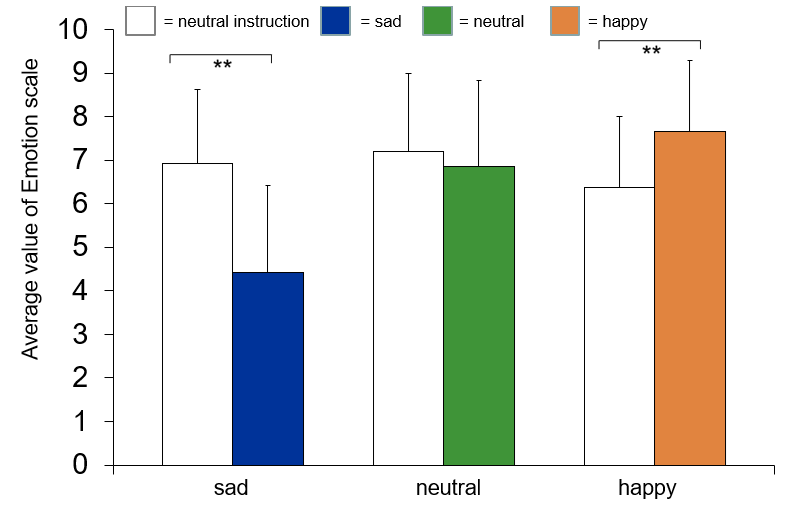


**Fig. S1.** **Comparison of mean values (+/- SE) of the self emotion rating of owners for the sad, neutral and happy stimuli, compared to the neutral instruction session.**

*Procedure*

Before the data collection started, the experiment was piloted with two owners and their dogs. After the test, participants received a little present (dog toy or dog treats or book voucher) as an expense allowance.

All participants were informed about their task with the following instructions:

“Please teach your dog to run around the cone in a certain time interval. You may verbally praise and stroke your dog, but do not use any reward in the form of treats. In the first round, show your dog to walk around the cone. When you feel your dog has understood this exercise, stop at the first line in front of the cone and have your dog "sit" at this line. Then send your dog around the cone. When your dog has done this three times, stand at the second line away from the cone and repeat the trick. It is important that you always stay at the appropriate line and that the dog sits before the exercise! You can always take a step towards the cone if it becomes too difficult for the dog. Repeat this exercise until the experimenter lets you know that the time is over.

Then give yourself and your dog a break. Your dog may lie down on the blanket, but it does not have to. During the break, the dog is allowed to move freely in the room. Please do not interact with him no matter what he does. This is important to make the break the same for all participants. To make it easier for you, sit down at the laptop and watch a video. The experimenter will start the video for you. Please put on your headphones for this. The break is over when the video ends.

Then get up, call your dog and start at the first line close to the cone and repeat the exercise.

If you have any questions, please contact the experimenter.”


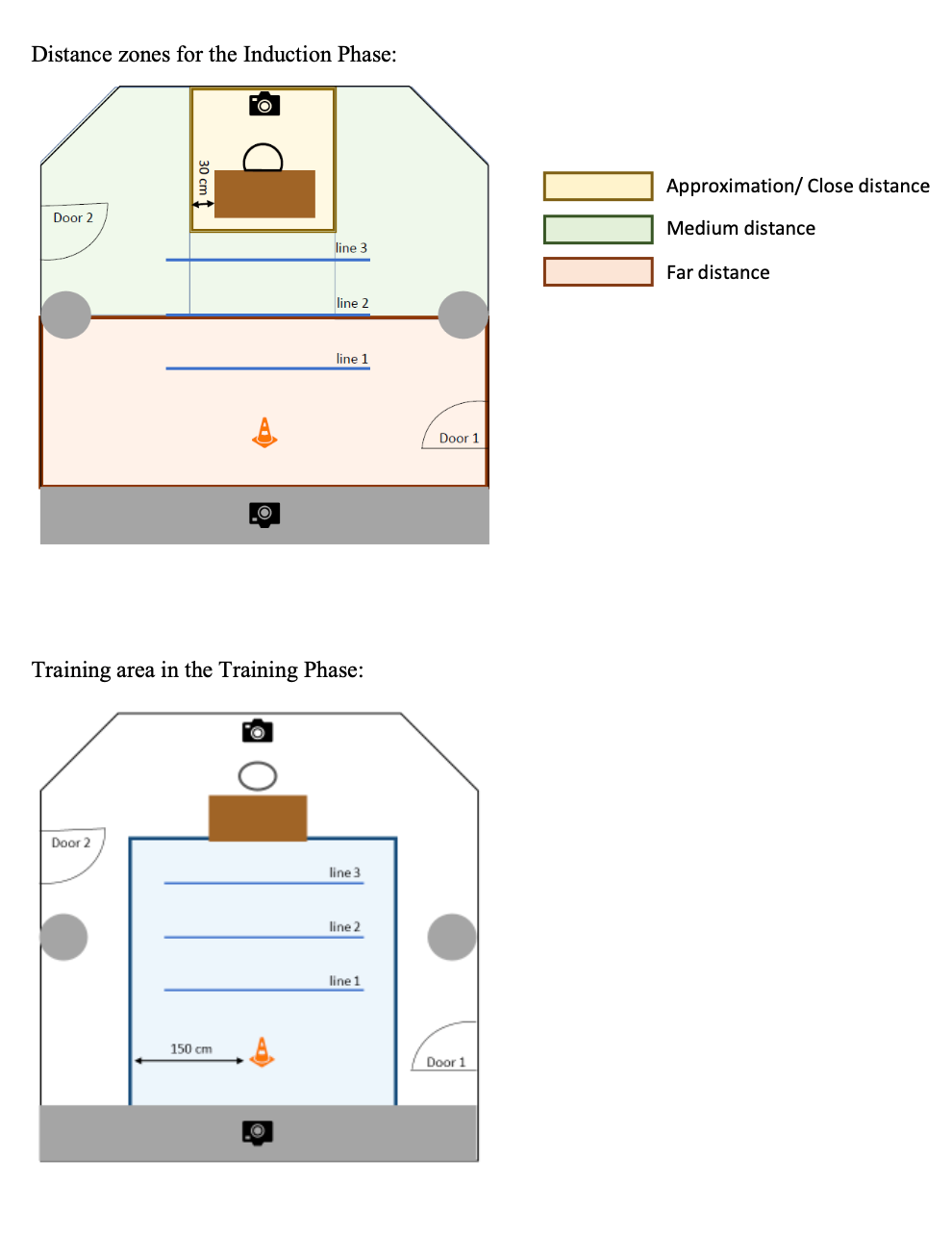
**Fig. S2.** **Detailed setup in the induction phases including the areas that were defined to code the distance between dog and owner.**

To code the clip induction phases, we defined the end of the phase as the point at which the owner removed the headphones. We started coding 120 seconds before this end point. We started coding the instruction induction phase when the experimenter had left the room and closed the door. The end of this phase was defined by the owner getting up. As it varied how long the owners read the instructions, we analysed the percentage (behavioural measures per time) in the induction phases (see also Table 1). We started coding the training phase when the owner got up after the induction phase - and we then coded the training for 180 seconds.

**Results**

**Table S2: Results of the Between-group comparison for the clip induction phase using the Kruskall-Wallis test.**

| Variable | Comparison | Coding | *M* | *SE* | *r* | *p* |
| --- | --- | --- | --- | --- | --- | --- |
| Gaze | Sad, happy, neutral | Frequency | Sad: 2,10  happy: 2.30  neutral: 2.69 | Sad: .43  happy: .58  neutral: .43 | .31 | .256 |
| Touch | Sad, happy, neutral | Frequency | Sad: .19  happy: .24  neutral: .45 | Sad: .07  happy: .09  neutral: .17 | .03 | .889 |
| Approach | Sad, happy, neutral | Frequency | Sad: .80  happy: .90  neutral: .96 | Sad: .13  happy: .20  neutral: .19 | 0 | .979 |
| Lay/sit | Sad, happy, neutral | Frequency | Sad: 60.45  happy: 55.75  neutral: 40.37 | Sad: 9.36  happy: 8.36  neutral: 7.71 | .32 | .243 |
| Distance far | Sad, happy, neutral | Frequency | Sad: 15.93  happy: 25.42  neutral: 23.70 | Sad: 5.59  happy: 7.26  neutral: 6.38 | .29 | .286 |

*Note.* Significant results are in bold.

**Table S3: Results of the Between-group comparison for the clip training phase using the Kruskall-Wallis test for continuous and the Pearson chi-square test (or fishers exact test) for categorical variables.**

| Variable | Comparison | Coding | *M* | *SE* | *r/v* | *p* |
| --- | --- | --- | --- | --- | --- | --- |
| Touch | Sad, happy, neutral | Frequency | Sad: 14.07  happy: 12.83  neutral: 15.58 | Sad: 2.07  happy: 1.79  neutral: 1.99 | *r* = .08 | .702 |
| Jump | Sad, happy, neutral | Occurrence | Sad: .30  happy: .33  neutral:.62 | Sad: .09  happy: .10  neutral: .10 | *v* = .29 | **.038** |
| Sit obeyed | Sad, happy, neutral | Frequency | Sad: 6.04  happy: 4.92  neutral: 6.92 | Sad: .80  happy: 1.04  neutral: .73 | *r* = .62 | .065 |
| Gaze | Sad, happy, neutral | Frequency | Sad: 24.67  happy: 21.63  neutral: 22.96 | Sad: 1.74  happy: 1.85  neutral: 1.63 | *r* = .23 | .365 |
| Overall success | Sad, happy, neutral | Frequency | Sad: 3.56  happy: 4.25  neutral: 2.50 | Sad: 1.07  happy: 1.91  neutral:.50 | *r* = 0 | .992 |

*Note.* Significant results are in bold.
